# Skin adnexal carcinoma of the eyelid: A case report

**DOI:** 10.3892/ol.2024.14811

**Published:** 2024-11-19

**Authors:** Rebaz H. Ali, Rawa M. Ali, Shano M. Ali, Ronak S. Ahmed, Ari M. Abdullah, Sami S. Omar, Hawkar A. Nasralla, Berun A. Abdalla, Sasan M. Ahmed, Fahmi H. Kakamad

**Affiliations:** 1 Hiwa Cancer Hospital Centre, Sulaymaniyah Directorate of Health, Sulaymaniyah, Kurdistan 46001, Iraq; 2 Department of Scientific Affairs, Smart Health Tower, Sulaymaniyah, Kurdistan 46001, Iraq; 3 Shahid Nabaz Dermatology Teaching Center for Treating Skin Diseases, Sulaymaniyah Directorate of Health, Sulaymaniyah, Kurdistan 46001, Iraq; 4 Department of Pathology, Sulaymaniyah Surgical Teaching Hospital, Sulaymaniyah, Kurdistan 46001, Iraq; 5 Rizgary Oncology Center, Erbil, Kurdistan 44001, Iraq; 6 Kscien Organization for Scientific Research, Sulaymaniyah, Kurdistan 46001, Iraq; 7 College of Medicine, University of Sulaimani, Sulaymaniyah, Kurdistan 46001, Iraq

**Keywords:** skin adnexal carcinoma, eyelid, chemotherapy, radiotherapy

## Abstract

Skin adnexal carcinomas (SACs) represent a diverse range of cancerous growths originating from the appendages of the skin. SACs are exceedingly rare malignancies that primarily manifest in individuals with fair skin and predominantly affect the head and neck. In the present study, a 70-year-old male presented with swelling and redness around the right eye, accompanied by skin desquamation. A tissue biopsy from the lesion showed a poorly differentiated SAC. Magnetic resonance imaging (MRI) of the neck and base of the skull revealed the presence of a large, well-defined heterogeneous mass involving the right orbit and periorbital tissue, measuring 62×38×43 mm. The patient declined wide local excision of the tumor. Eventually, the patient received a combination of chemotherapy and radiotherapy, followed by additional cycles of chemotherapy. Then, 1 month after the final chemotherapy, an MRI showed a small, linear lesion (8×4 mm) in the lower right orbital cavity, reduced by 90% from its original size. The scan also revealed diffuse volume loss and irregularity in the right eye, likely due to radiation, leading to vision loss in the affected eye. At present, the exact cause of SACs remains largely unidentified. While SACs frequently develop spontaneously, some may originate from precursor lesions or preexisting benign counterparts. The incidence of SAC is exceedingly rare, particularly when it is found in the eyelid. A combination of chemotherapy and radiotherapy followed by additional cycles of chemotherapy may be an effective therapeutic modality in shrinking the tumor.

## Introduction

Skin adnexal carcinomas (SACs) represent a diverse range of cancerous growths originating from the appendages of the skin. According to the World Health Organization categorization of skin carcinomas, adnexal adenocarcinoma of ‘not otherwise specified’ type is a primary skin adenocarcinoma characterized by ductal or glandular differentiation, lacking distinct histological features for more specific classification (1).

SACs are exceedingly rare malignancies (2,3), and exhibit diverse patterns and differentiations, with over 15 distinct histologies. These carcinomas stem from three different structures: The pilosebaceous unit, the eccrine sweat glands and the apocrine glands. Due to their rarity and challenges in recognition, coupled with their potential aggressive behavior, treating SACs poses a significant challenge (4,5).

Primarily, SACs manifest in individuals with fair skin, predominantly affecting the head and neck (65%), followed by the extremities (17%) and trunk (17%). However, the incidence of SAC increases significantly with advancing age, reaching its peak during the eighth decade of life (6,7). These tumors have also been documented in different ethnic groups, including African-American, Asian and Pacific Islander individuals (8,9). Typically, SACs present as slowly growing masses, appearing either skin-colored or red. In most cases, SACs are asymptomatic and rarely induce itching, bleeding or pain. While most SACs exhibit only local aggressiveness, they possess the capability for regional and distant metastasis (10). The exact cause of SACs remains unidentified. Certain factors, such as ultraviolet radiation, immunosuppression, organ transplant and genetic disorders, may play a role (10–13).

The present study describes a rare case of adnexal carcinoma of no specific differentiation in the right eyelid treated with a combination of chemotherapy and radiotherapy. The case report aimed to avoid citing predatory publications based on Kscien's list (14).

## Case report

### Patient information

**Figure 1.**
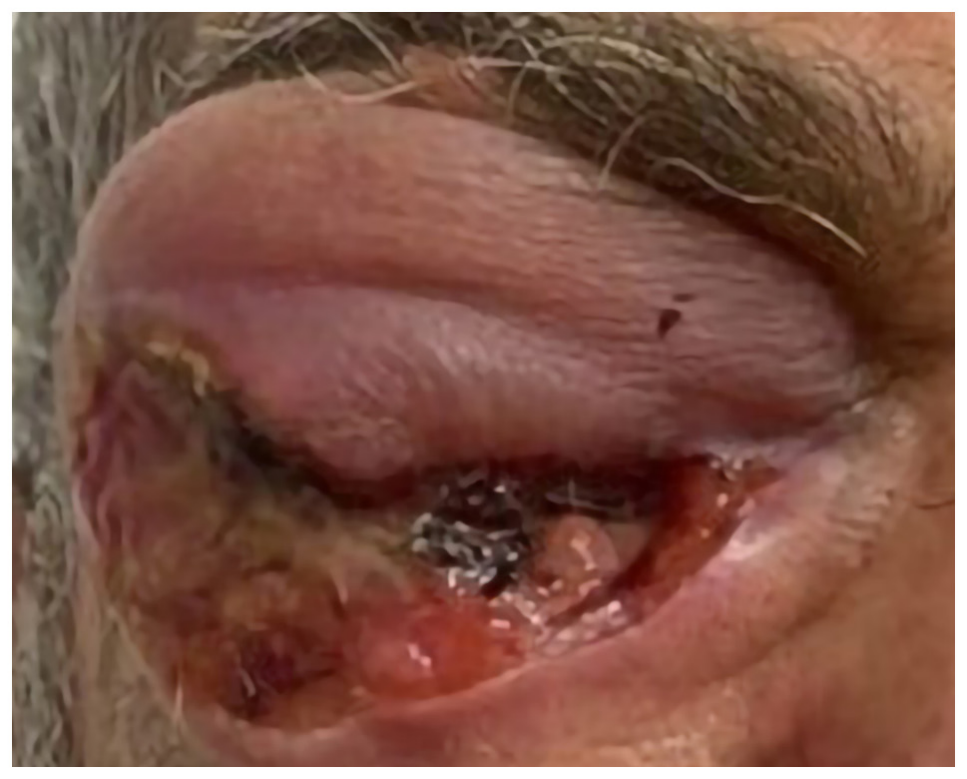
Skin adnexal carcinoma affecting the right eye (pre-treatment appearance).

A 70-year-old male with fair skin presented to Smart Health Tower (Sulaymaniyah, Iraq) in December 2023 with swelling and redness around the right eye, extending to cover the eye, along with skin desquamation (Fig. 1). Based on the description from the patient, the lesion first appeared as a minor redness around the eye nearly 19 years ago. However, it is unlikely that the current lesion was the same one due to its long existence. It is possible that the patient might have mistaken the timeline or the lesion could be a different benign skin growth. In recent years, the lesion recurred intermittently and developed slight scaling over time. Despite topical treatments, the lesion persisted and eventually grew in size, forming a wound prone to bleeding. Notably, there was no family history of malignancies, but the patient had a history of excessive sunlight exposure. The patient had twice undergone an inguinal hernia repair, first in 2002 and then again in 2010. Prior to arriving at Smart Health Tower, the patient visited the Oncology Teaching Hospital (Baghdad, Iraq), in May 2023 for the same condition. A histopathological examination was carried out (please see below) at the National Center for Educational Laboratories (Baghdad, Iraq). Following the diagnosis, the patient decided to seek treatment at Smart Health Tower.

### Clinical findings

All vital signs were within the normal ranges. An ulcer with swelling involving the right lower eyelid was apparent. The patient's vision in the right eye was obstructed by swelling that covered the eye.

### Diagnostic approach

**Figure 2.**
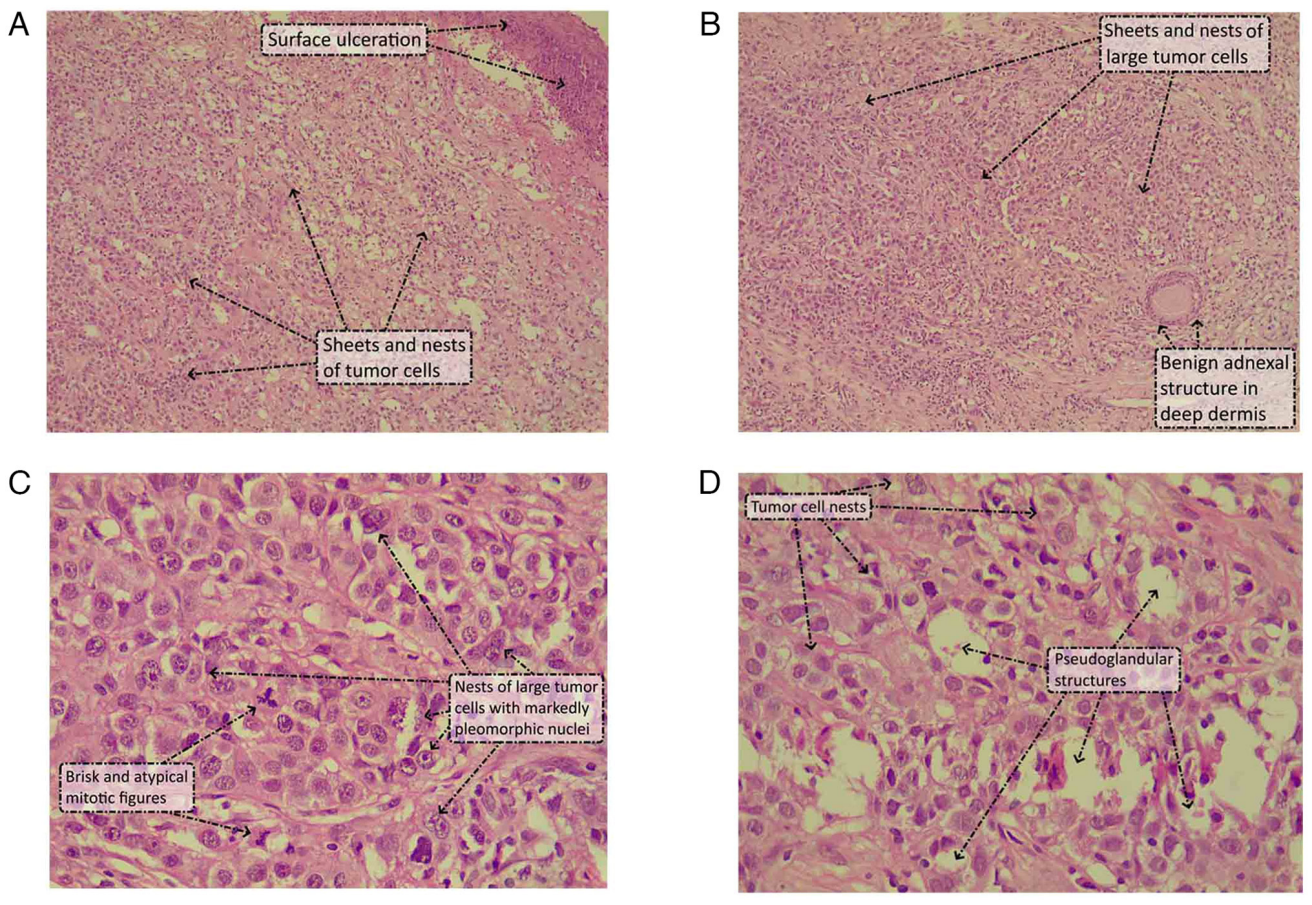
Hematoxylin and eosin-stained images of the tumor biopsy. (A) The tumor cells were arranged in sheets and nests underneath the ulcerated epidermis (magnification, ×40). (B) Sheets and nests of large tumor cells infiltrated deeply around the benign adnexal structures (magnification, ×40). (C) The tumor cells were arranged in nests and had an abundant lightly eosinophilic to clear cytoplasm with markedly pleomorphic, large nuclei that had vesicular chromatin, prominent eosinophilic nucleoli and irregular nuclear outlines. There was brisk mitosis with atypical (tripolar) mitotic figures (magnification, ×400). (D) The tumor cells were predominantly arranged in nests with occasional pseudoglandular structures (magnification, ×400).

Before the patient presented at Smart Health Tower, an incisional biopsy was performed in May 2023 at the Oncology Teaching Hospital, followed by a histopathological examination at the National Center for Educational Laboratories (Baghdad, Iraq). The hematoxylin and eosin-stained sections showed a skin biopsy with a proliferation of cells underneath an ulcerated epidermis extending to the dermis and around the benign adnexal structures. The tumor cells were arranged in sheets, nests and occasional pseudo-glandular structures. The cells were large and had an abundant lightly eosinophilic to clear cytoplasm, with markedly pleomorphic, large nuclei that had vesicular chromatin, prominent eosinophilic nucleoli and irregular nuclear outlines. There was brisk mitosis with atypical (tripolar) mitotic figures. There was no squamous, apocrine, sebaceous or any other form of distinct histogenetic differentiation in any area of the tumor in the incisional biopsy (Fig. 2). A panel of immunohistochemical stains was performed on the biopsy, which showed positive reactivity of the tumor cells to pan-keratin (AE1/AE3), cytokeratin (CK)7, vimentin and CD15. Stains for CK20, Melan-A, human melanoma black-45, S100, gross cystic disease fluid protein 15 and carcinoembryonic antigen were all negative. The combined histological and immunohistochemical picture supported the diagnosis of a poorly differentiated SAC with no specific histogenetic line of differentiation.

**Figure 3.**
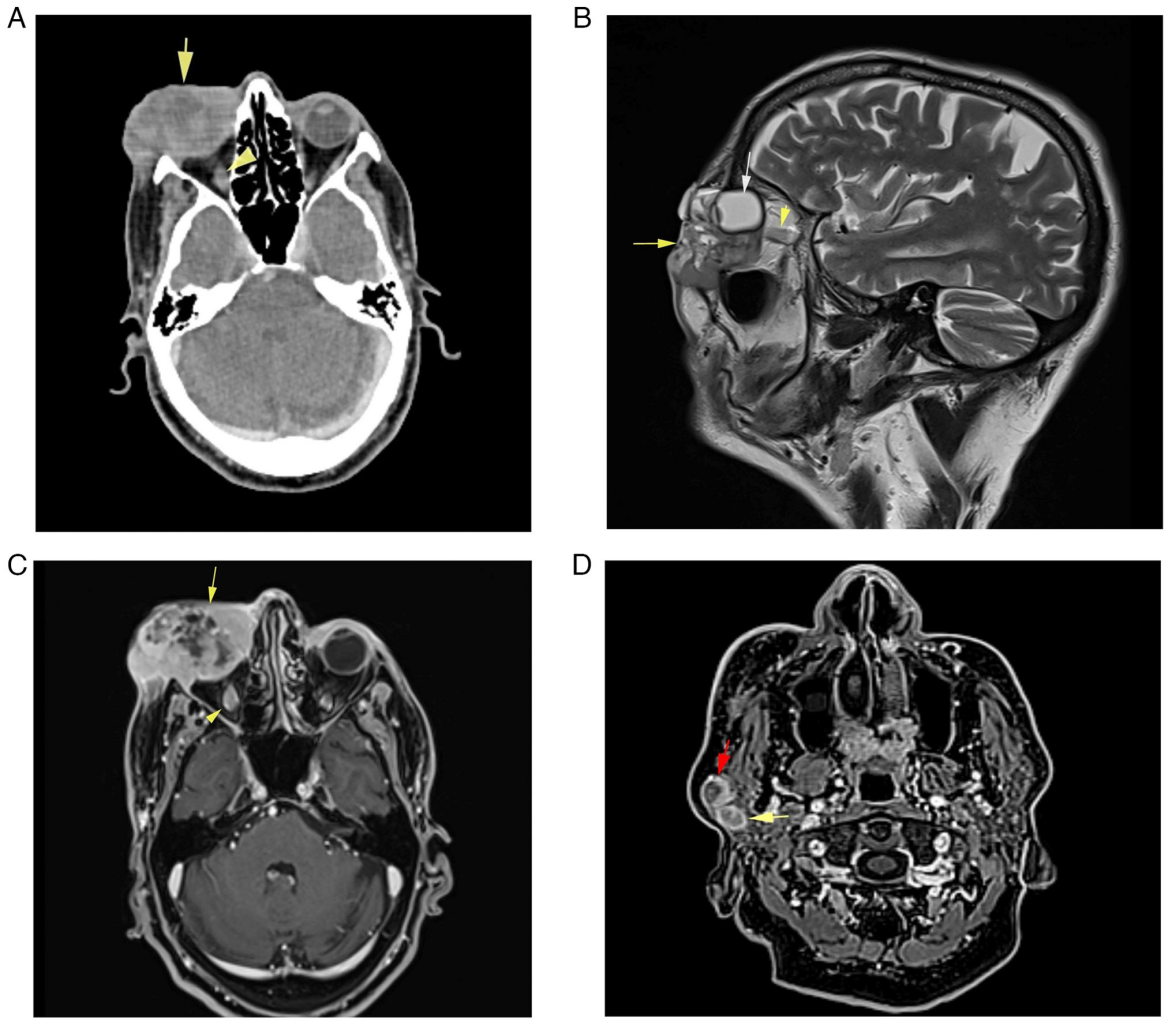
Pre-treatment imaging revealing a significant right periorbital mass. (A) An axial contrast-enhanced computed tomography scan of the face and skull exhibited a large, well-defined mass (arrow) and the optic nerve (arrowhead). (B) A sagittal T2-weighted image revealed a large cystic-solid lesion (yellow arrow) with orbital (white arrow), periorbital and intraorbital extension. The optic nerve appeared free of any invasion (yellow arrowhead). (C) Post-contrast axial T1 fast suppression imaging showed a large, enhanced lesion (arrow) with foci of cystic degeneration represented by non-enhancing areas and the optic nerve (arrowhead). (D) Pre-treatment axial fat-suppressed T1-weighted images revealed two oval-shaped lymph nodes within the parotid gland, exhibiting peripheral enhancement and central necrosis (yellow and red arrows).

Subsequent imaging studies conducted at Smart Health Tower included a computed tomography (CT) scan of the head and neck, revealing an ill-defined, heterogeneously enhancing soft-tissue mass occupying the skin, lateral canthus, right lacrimal gland, lower eyelid and preseptal space, measuring 44×54×32 mm (Fig. 3A). This mass extended to the medial canthus and intraconal space, infiltrating the lateral and inferior recti muscles of the right eye. No calcification or bone destruction was observed. Furthermore, multiple right intraparotid lymph nodes with rounded contours and central necrosis, the largest measuring 16×15 mm, were noted, indicative of lymph node infiltration (Fig. 3D).

**Figure 4.**
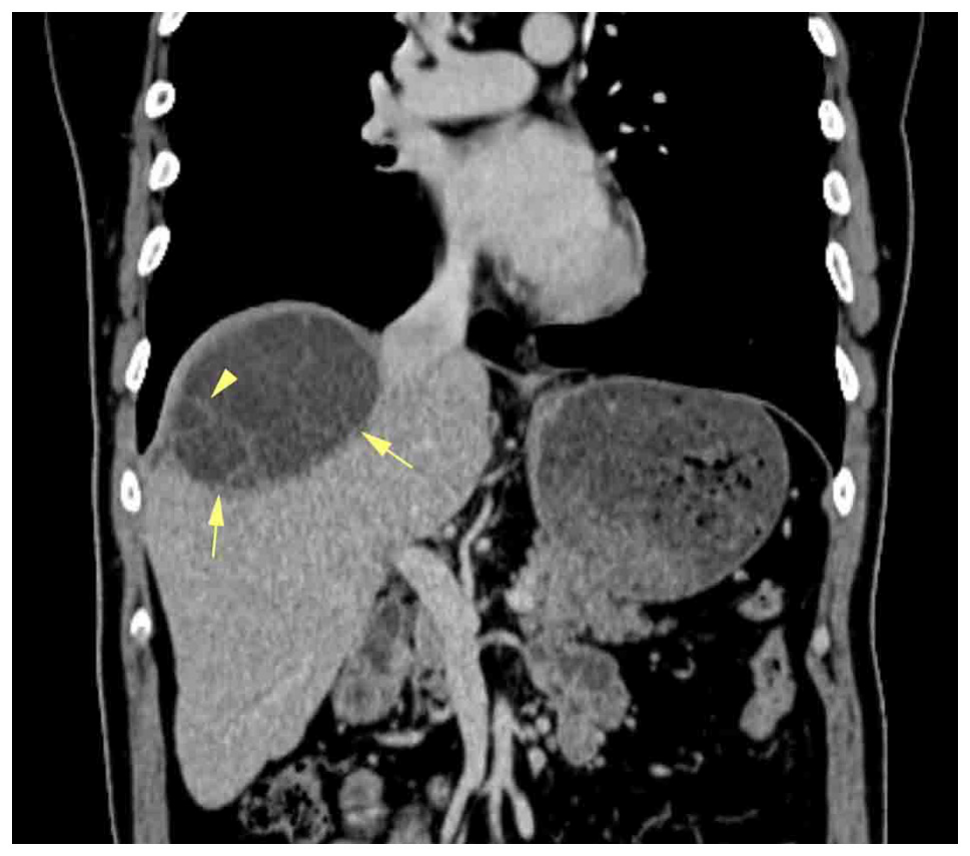
A coronal reformatted computed tomography scan revealed a large, thick-walled subcapsular cystic lesion (arrows) with multiple thin internal septations (arrowhead), located at the liver dome just beneath the right hemidiaphragm.

Magnetic resonance imaging (MRI) of the neck and base of the skull with contrast revealed the presence of a large, well-defined heterogeneous mass involving the right orbit and periorbital tissue, measuring 62×38×43 mm (Fig. 3). The tumor partially encroached upon the right orbit and the right lacrimal gland, with invasion noted into the peri-orbital muscles but excluding the superior rectus muscle. There was no evidence of bone invasion or extension into the paranasal sinuses, and the integrity of the optic nerve was preserved. Incidentally, mild involutional brain changes were observed. Furthermore, two pathological lymph nodes measuring <14 mm were detected within the right parotid gland. A CT scan of the chest and abdomen revealed multiple cystic lesions in the liver, with the largest measuring 73×70 mm in segment VII. These lesions appeared multilocular, containing multiple enhanced septa suggestive of multiple hydatid cysts (Fig. 4).

### Therapeutic intervention

**Figure 5.**
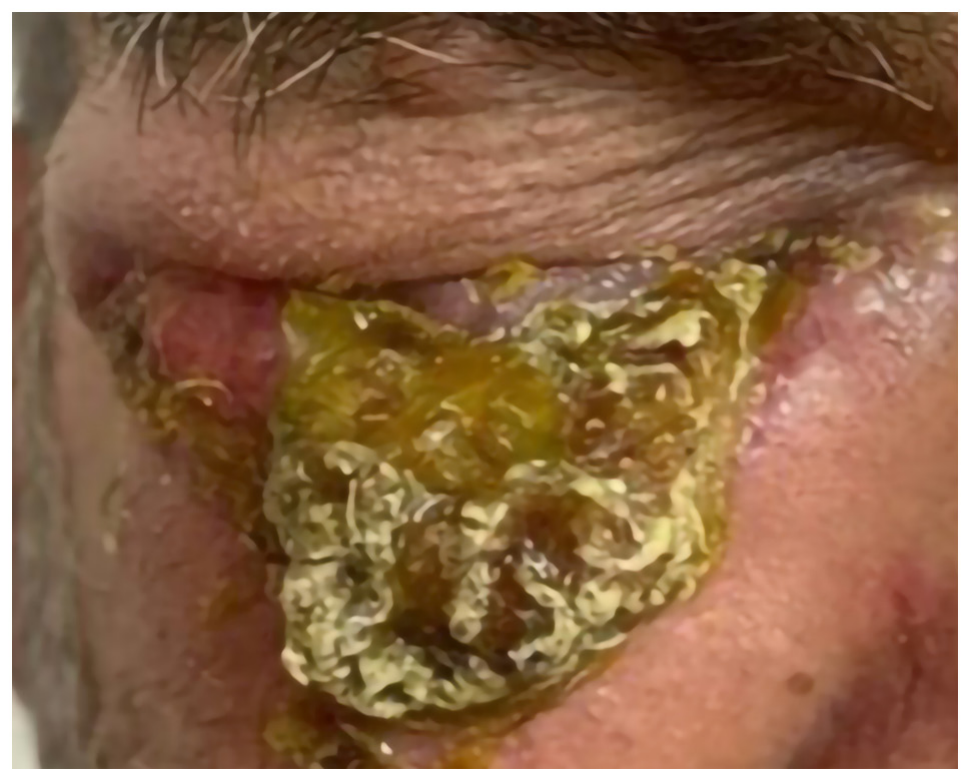
Decreased swelling and ulceration around the affected eye during treatment.

The patient refused any form of surgical intervention for the tumor apart from the incisional biopsy. Chemotherapy consisting of carboplatin and paclitaxel was administered weekly for 6 weeks, employing a regimen of carboplatin with an area under the curve (AUC) value of 2 in conjunction with paclitaxel dosed at 50 mg/m^2^, followed by curative radiotherapy utilizing volumetric modulated arc therapy. The radiation was focused on the primary target volume (PTV)1, encompassing the right cervical regions 2 and 3 and the entire parotid gland, with a cumulative dose of 5,940 cGY delivered over a total of 33 fractions spanning 33 days; this led to a reduction in swelling and ulceration around the affected eye (Fig. 5). Additionally, a PTV2 covering the eye and including the gross tumor volume of the lymph nodes within the intraparotid region, with a total dose of 6,996 cGY, was administered over the same 33 fractions and days. After this combined modality therapy, the patient underwent an additional three cycles of carboplatin and paclitaxel chemotherapy administered at intervals of 21 days. The paclitaxel was dosed at 175 mg/m^2^, while carboplatin was capped at a maximum of 750 mg, with a target AUC of 5.

### Follow-up and outcome

**Figure 6.**
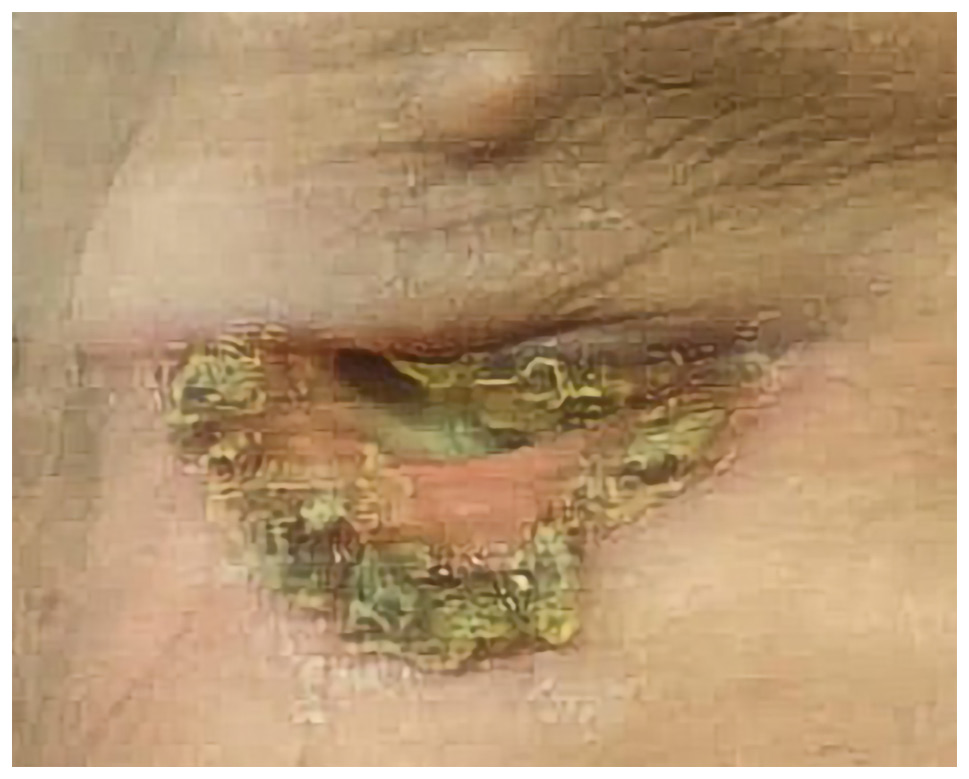
Post-treatment results after combining chemotherapy and radiotherapy, followed by additional chemotherapy cycles. A notable reduction in tumor size was observed, shrinking from an initial 62 to 8 mm in diameter.

**Figure 7.**
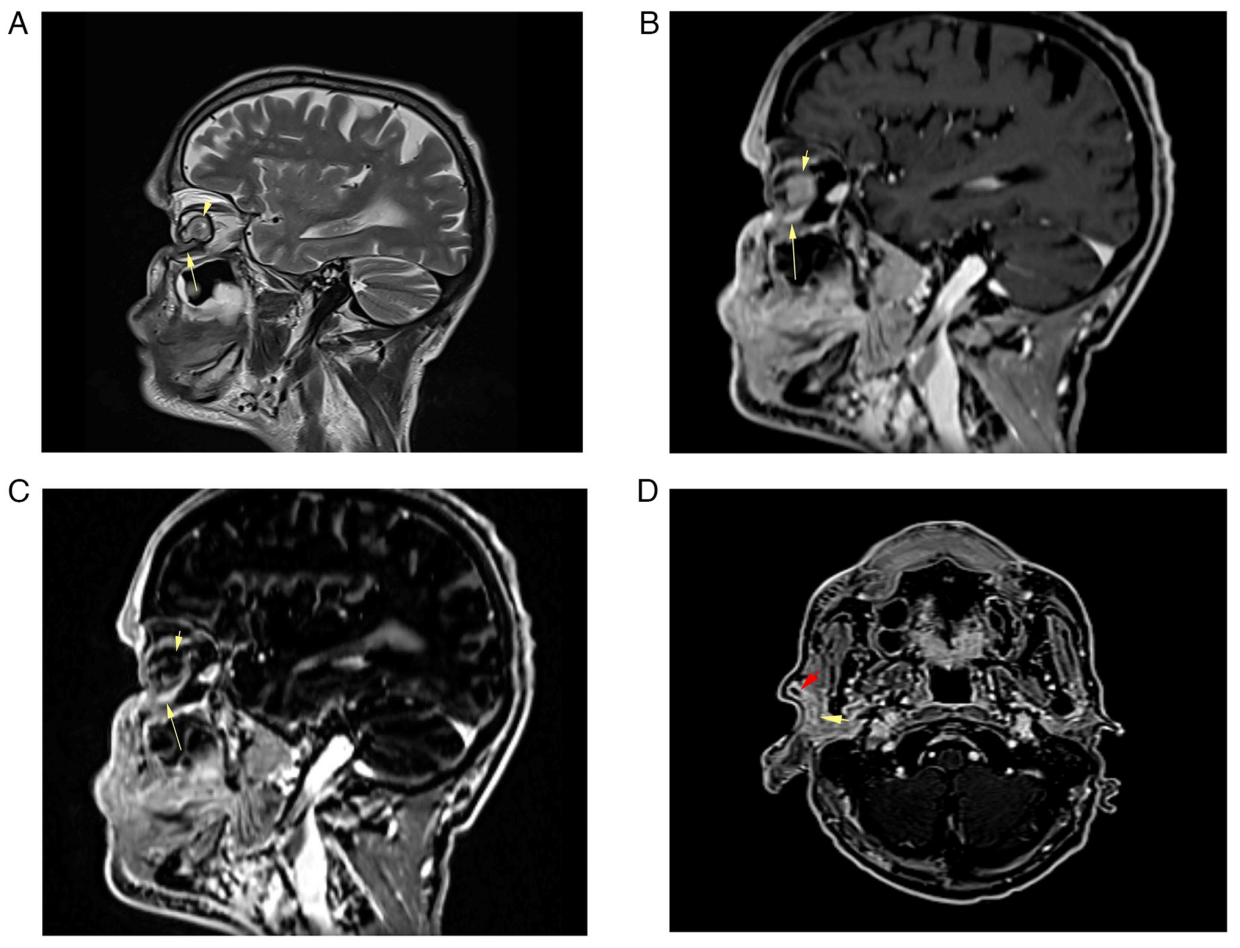
Post-treatment magnetic resonance imaging showing a significant reduction (~90%) in the tumor size. (A) A sagittal T2-weighted image showing a shrunken intraorbital mass (arrow) with atrophy of the right orbit, resulting in phthisis bulbi, which contained a hemorrhagic component (arrowhead). (B) A sagittal post-contrast fat-suppression T1-weighted image showing an infraorbital linear enhancing component measuring 8×4 mm, with the intraorbital homogenous component appearing hyperintense (arrowhead). (C) A sagittal subtraction sequence showing an enhancing infraorbital linear focus (arrow), representing residual tumor after chemotherapy, with the intraorbital hemorrhagic component showing no post-contrast enhancement (arrowhead). (D) Post-treatment axial fat-suppressed T1-weighted images demonstrated a significant reduction in the size of both lymph nodes (yellow and red arrows).

Upon follow-up, 1 month after the last chemotherapy (Fig. 6), an MRI revealed a small, linear-shaped focal enhancing lesion measuring 8×4 mm in the inferior segment of the right orbital cavity. This lesion showed a significant reduction from its initial size of 62×43×38 mm, indicating 90% post-treatment shrinkage (Fig. 7). According to the Tumor-Node-Metastasis (TNM) classification (8th edition) (15), the tumor was initially classified as T4 due to its size and involvement of the orbit and periorbital tissues. Following treatment, the significant reduction in tumor size led to a down-staging to T1.

Additionally, diffuse volume loss and irregularity in the right eyeball were observed, suggesting radiation-induced changes, which resulted in vision loss in the affected eye. The enlarged lymph nodes in the right cervical region, previously displaying suspicious imaging features, exhibited a reduction in size of ~40% relative to their prior assessments (Fig. 7). The patient was advised on the option of surgery for the residual tumor but declined the treatment. No further treatment was administered to the patient, and the patient is scheduled for follow-up every 3 months.

## Discussion

Cutaneous adnexal carcinomas are rare, accounting for only 0.005% of all skin tumors (16). SACs typically exhibit gradual growth and appear either red or skin-colored. Detection usually occurs after the lesion has been present for an extended period, as SACs are commonly symptomless. Although infrequent, SACs may occasionally cause itching, bleeding or discomfort. SACs are predominantly found in individuals with fair skin, mainly on the head and neck region (6,10). The patient in the present case report was a fair-skinned male. According to the description provided by the patient, the lesion was first observed 19 years ago as a small red area around the right eye, which raises some uncertainty. Over time, the lesion intermittently diminished and regrew multiple times. Despite attempts with topical treatments, the lesion endured and eventually grew in size, evolving into a wound susceptible to bleeding.

The exact cause of SACs remains largely unidentified. While SACs frequently develop spontaneously, some may originate from precursor lesions or preexisting benign counterparts. Malignant transformation within benign adnexal skin tumors has been documented, particularly in immunosuppressed patients (11). Moreover, organ transplant recipients demonstrate a propensity for developing SACs (10). The presence of a genetic basis is observed in various syndromes linked to multiple adnexal tumors and systemic malignancies, such as sebaceous carcinoma in Muir-Torre syndrome and cylindrocarcinoma in Brooke-Spiegler syndrome (12,13). Ultraviolet radiation is considered a potential etiological factor for SACs, similar to its role in other types of skin cancer. This is supported by the tendency of SACs to predominantly affect the head and neck region, where exposure to UV radiation is often more marked (10). The patient described in the present study had a history of excessive sunlight exposure, but no known family history, syndromic manifestations or organ transplantation.

A study involving 23 cases of SAC found that the median age at diagnosis was 66 years (range, 46–87 years), with 61% of patients being female and 39% male (5). Another population-based study conducted by Stam *et al* (10) in the Netherlands, involving 2,220 patients with SACs, revealed that 52.70% of patients were female and 47.30% were male. Most SACs occurred in the head and neck region. Notable occurrences were observed in areas such as the eyelid (9%), ear (7% for males and 3% for females) and lip (2%). Other affected regions included the trunk (19%), extremities (15%) and genitals (14%). The patient described in the present case report was a 70-year-old male.

Clinically diagnosing SACs is challenging even for experienced skin cancer specialists, as they are often mistaken for squamous cell carcinoma, basal cell carcinoma or benign skin tumors. This challenge in identifying SACs clinically can be attributed to their diverse and frequently subtle manifestations (5,17,18). The histopathological assessment of a deep skin biopsy or diagnostic excision is widely accepted as the primary method for diagnosing SAC. Nevertheless, due to the diverse histopathological characteristics of SACs, achieving an accurate histological diagnosis can frequently pose challenges (6,18). In the present case report, a skin tissue biopsy was obtained for histopathological examination, revealing histological features consistent with poorly differentiated adnexal skin carcinoma, but without any specific features to suggest squamous, apocrine, sebaceous or other distinct histogenetic lines of differentiation.

Due to the scarcity of SACs, consensus regarding their management is lacking, leading to treatments typically based on limited case series. Surgery, commonly involving wide local excision or Mohs surgery, has been the predominant approach for most cases (7,9,19,20). The patient in the present case report declined to undergo a wide local tumor excision. Reports indicate that local or regional recurrence occurs in up to 60% of lesions treated solely with surgical excision for cutaneous adnexal carcinomas (4). The infiltrative nature of these lesions often complicates the process of obtaining sufficient margins without substantial surgical defects. Surgical series have documented a re-excision rate of up to 30% among patients who underwent wide local excision alone due to persistently positive margins (19,21).

Additionally, the literature presents mixed outcomes regarding the efficacy of radiation therapy as a standalone treatment. In one case report, radiation alone was administered for a lower lip lesion alongside a clinically positive submental lymph node. After a 6-month follow-up, the patient exhibited no signs of disease (22). However, transformation into a more aggressive histological form has also been documented in a case where radiation was used as the sole treatment (23). The present case report documents the administration of chemotherapy and radiotherapy followed by additional cycles of chemotherapy without surgery. Notably, the tumor showed a marked reduction in size of >90%, while the two suspicious right cervical lymph nodes exhibited a reduction in size of ~40%. According to the TNM classification (8th edition), the tumor was initially classified as T4 due to its size and involvement of the orbit and periorbital tissues. After treatment, the marked reduction in tumor size indicated a down-staging to T1 (15). This substantial shift from T4 to T1 underscores the effectiveness of combined chemotherapy and radiotherapy in reducing the tumor burden, thereby making curative surgery a viable option. However, the patient opted against this surgery.

The limitation of the present report is the inability to assign a specific histotype to the SAC. This is due to the patient declining a larger tissue biopsy and surgical removal of the mass to allow a more complete assessment of the entire lesion. The available immunohistochemical staining results supported the overall diagnosis, but more niche and experimental stains were not available to favor one line of differentiation over another. Furthermore, molecular testing and genomic profiling studies are not readily available in Iraq to perform on the biopsy material.

In conclusion, SAC is a rare finding and its occurrence in the eyelid is even rarer. A combination of chemotherapy and radiotherapy followed by additional cycles of chemotherapy can be an effective therapeutic modality in minimizing the size of the tumor.

## Data Availability

The data generated in the present study may be requested from the corresponding author.
